# Diversity, Distribution and Structural Prediction of the Pathogenic Bacterial Effectors EspN and EspS

**DOI:** 10.3390/genes15101250

**Published:** 2024-09-26

**Authors:** Zhan Li, Yuru Hu, Yuan Song, Deyu Li, Xiaolan Yang, Liangyan Zhang, Tao Li, Hui Wang

**Affiliations:** 1 State Key Laboratory of Pathogens and Biosecurity, Academy of Military Medical Sciences, Beijing 100071, China; yexi19881214@126.com (Z.L.); huyururu@126.com (Y.H.); songyuanzgsd@163.com (Y.S.); 18842345409@163.com (D.L.); yangxiaolanyxl@163.com (X.Y.); polini@live.cn (L.Z.); 2 School of Basic Medical Science, Anhui Medical University, Hefei 230032, China

**Keywords:** enterobacteria, distribution, structure prediction

## Abstract

Background: Many Gram-negative enterobacteria translocate virulence proteins (effectors) into intestinal epithelial cells using a type III secretion system (T3SS) to subvert the activity of various cell functions possess. Many T3SS effectors have been extensively characterized, but there are still some effector proteins whose functional information is completely unknown. Methods: In this study, two predicted effectors of unknown function, EspN and EspS (*Escherichia coli* secreted protein N and S), were selected for analysis of translocation, distribution and structure prediction. Results: The TEM1 (β-lactamase) translocation assay was performed, which showed that EspN and EspS are translocated into host cells in a T3SS-dependent manner during bacterial infection. A phylogenetic tree analysis revealed that homologs of EspN and EspS are widely distributed in pathogenic bacteria. Multiple sequence alignment revealed that EspN and its homologs share a conserved C-terminal region (673–1133 a.a.). Furthermore, the structure of EspN (673–1133 a.a.) was also predicted and well-defined, which showed that it has three subdomains connected by a loop region. EspS and its homologs share a sequence-conserved C-terminal (146–291 a.a.). The predicted structure of EspS (146–291 a.a.) is composed of a β-sheet consisting of four β-strands and several short helices, which has a TM score of 0.5014 with the structure of the *Vibrio cholerae* RTX cysteine protease domain (PDBID: 3eeb). Conclusions: These results suggest that EspN and EspS may represent two important classes of T3SS effectors associated with pathogen virulence, and our findings provide important clues to understanding the potential functions of EspN and EspS.

## 1. Introduction

Many Gram-negative enterobacteria, such as *Escherichia*, *Shigella*, *Salmonella*, and *Citrobacter*, can cause many serious infections. Two of the enteric *E. coli* pathotypes, enteropathogenic *E. coli* (EPEC) and enterohaemorrhagic *E. coli* (EHEC) are important foodborne pathogens that can cause serious complications, including hemorrhagic colitis and hemolytic uremic syndrome and contribute significantly to morbidity and mortality worldwide [1,2,3]. Their murine counterpart, *Citrobacter rodentium*, is an extracellular enteric mouse-specific pathogen used to model infections with human pathogenic *E. coli* and inflammatory bowel disease [4]. *Salmonella* and *Shigella* are known to cause a well-characterized spectrum of diseases in humans, ranging from asymptomatic carriage to hemorrhagic colitis and fatal typhoidal fever [5,6]. These pathogens have acquired virulence effectors that enable them to colonize, multiply, and evade host defenses. The best-characterized system for delivering effector proteins into target eukaryotic cells is the type III secretion system (T3SS), which is expressed by Gram-negative bacteria [7]. The structural component of the T3SS is the needle apparatus, which is assembled from over 20 different proteins and consists of a base, an extracellular needle, a tip, and a translocon [8]. The T3SS is essential for colonization and pathogenesis, and mutants lacking this apparatus fail to cause disease [9].

The T3SS is used to translocate dozens to hundreds of bacterial effector proteins directly into the host cytosol, where they manipulate a variety of host cell processes to establish a successful infection [10]. Many of these translocated proteins have been described in pathogenic *E. coli* strains (EHEC, EPEC, etc.). A number of T3SS effectors, such as non-LEE effectors (Nle) [11], are important mediators of virulence. NleH and NleD are inhibitors of cell apoptosis [12]. While, NleB, NleC, and NleE are involved in the interference with host inflammatory signaling pathways [3]. NleB, a bacterial effector with glycosyltransferase activity, targets GAPDH function to inhibit NF-κB activation [13]. NleC, a type III secretion protease, compromises NF-κB activation by targeting p65/RelA [14], and NleE inhibits NF-κB activation [15]. The *E. coli* secreted proteins (Esp) [16] also play an important role in infection. For example, EspH inactivates multiple host Rho GEFs through binding to their Dbl-homology and pleckstrin homology (DH-PH) domain, preventing Rho GTPase activation [7]. EspF coordinates the spatiotemporal activation of two eukaryotic signaling pathways, thereby regulating membrane trafficking [17].

In addition to the effectors described above, numerous other T3SS effectors have been identified by genome sequencing for which no functional information is currently available, such as EspN and EspS. The *espN* gene was initially identified in *E. coli* O157:H7 strain EDL933, which is a potential T3SS effector with an unconfirmed function [18]. The *espN* gene is located on the top strand from 1582242 to 1585643. The EspN protein is composed of 1133 amino acids. Additionally, the *espS* gene was identified in *E. coli* O145:H28 RM13514 as a T3SS effector with an unknown function [19]. The *espS* gene is located on the top strand from 3360604 to 3361479. The *espS* gene encodes a protein of 291 amino acids.

In this study, to confirm whether EspN or EspS is a T3SS effector, we performed a TEM1 (β-lactamase) transport assay, which indicated that EspN or EspS is transported into host cells in a T3SS-dependent manner. To further clarify the diversity and distribution of these two T3SS effectors, we performed an evolutionary tree analysis, which showed that homologous proteins of EspN and EspS are widely distributed in multiple pathogens. Then, to reveal the functional information of EspN and EspS, we predicted the 3D structures of EspN and EspS and compared them with those of known functional proteins, providing important clues to the functions of the two effectors. 

## 2. Materials and Methods

### 2.1. T3SS Translocation Assay

The genes *map-tem1*, *espN-tem1*, *espS-tem1*, or *tem1* were synthesized and inserted into expression vector pET-28a to construct TEM1 fusion expression plasmid, pMap-TEM1, pEspN-TEM1, pEspS-TEM1, or pTEM1 [20]. The expression plasmids pMap-TEM1, pEspN-TEM1, pEspS-TEM1, or pTEM1 were electrotransformed into EHEC (*E. coli* O157:H7 or *E. coli* O145:H28) wild-type or Δ*escN* strains whose expression was induced by IPTG, and prepared for a fluorescence-based reporter (TEM-1 β-lactamase) [21]. Human Colorectal Adenocarcinoma Cell Line HT-29 cells (ATCC HTB-38) were maintained in Dulbecco’s modified Eagle’s medium without antibiotics containing 10% fetal bovine serum at 37 °C in an atmosphere containing 5% CO_2_ [22]. HT-29 cells were infected with EHEC wild-type or Δ*escN* strains expressing different TEM-1 fusion proteins (Map-TEM1, TEM1, EspN-TEM1, or EspS-TEM1) at a MOI of 10 bacteria per cell. After 1 h of infection, IPTG was added to a final concentration of 1 mM, and the infection was allowed to proceed for an additional 3 h. Cell monolayers were then washed twice with Hanks Balanced Salt Solution (HBSS) and loaded with the fluorescent β-lactamase substrate CCF2/AM [23] according to the manufacturer’s instructions using a LiveBLAzer FRET-B/G loading kit (Thermo). The absence of translocated TEM1 resulted in the development of green fluorescence at 520 nm, which was attributed to CCF2/AM that had been loaded into the cells. Conversely, the blue fluorescence (at 450 nm), which was generated by β-lactamase-mediated cleavage of CCF2/AM, served as an indicator of translocated TEM1 fusion proteins. EspN or EspS translocation was examined on a confocal fluorescence microscope to detect the blue fluorescence (~450 nm) and green fluorescence emission (~520 nm) at 410 nm excitation (Olympus FV1000).

### 2.2. Bioinformatics Analyses

We first used the blastp (BLAST+ 2.15.0) tool [24] to perform sequence similarity comparison on the UniProt library (https://www.uniprot.org/, Uniprot release 2024_04, accessed on 31 July 2024) [25] using EspN and EspS sequences as query sequences. We used the default parameters of the blastp program, except that the evalue threshold was adjusted to 0.0001 to ensure sequence homology, and max_target_seqs was set to 10,000 to obtain as many sequences as possible. We further filtered the search results and retained sequences with the percentage of identical matches greater than 30 and query coverage per subject greater than 90. We found 441 sequences for EspN and 248 sequences for EspS. We then manually filtered the results and deleted highly duplicated sequences and those that had been deleted from the uniprot database. Finally, we retained 114 sequences for EspN and 69 sequences for EspS, respectively, for phylogenetic tree analysis.

### 2.3. Phylogenetic Analyses

To construct the phylogenetic tree, we used a dynamic programming algorithm to find the best global alignment between the two sequences. The number of matches divided by the length is the similarity score between the two sequences. We performed this calculation on the filtered sequences mentioned above to obtain the similarity matrix for EspN and EspS, respectively. One-minus the similarity matrix is the distance matrix. Then, we used the Unweighted Pair Group Method with the Arithmetic Mean (UPGMA) method [26,27] to calculate the phylogenetic tree. UPGMA is a hierarchical clustering analysis method that finds the two nearest samples or sample groups based on the distance matrix and merges them into a new group, then updates the average distance between this new group and other samples or groups, and then repeatedly finds the two nearest samples or sample groups and merges them until all samples are merged into one group. We used the ETE3 [28] python package to draw the phylogenetic tree. Sample groups with a distance greater than 0.1 are marked with different colors.

### 2.4. Multiple Alignment of EspN and Its Homologs, as Well as EspS and Its Homologs

The amino acid sequences of EspN and its homologs, as well as EspS and its homologs, were obtained from the Uniprot database (https://www.uniprot.org/, Uniprot release 2024_04, accessed on 31 July 2024) in FASTA format. Multiple alignments of these effectors were compared and aligned using BioEdit 7.7 [29] and ClustalW 7.7 software [30].

### 2.5. Protein Structure Prediction and Analysis

The 3D structures of EspN and EspS were generated using Alphafold2 [31] based on the protein sequences. For AlphaFold2, the default process and parameters are utilized for protein structure prediction, which involves the initial execution of Multiple Sequence Alignment (MSA), followed by End-to-End (E2E) inference, and finally, the application of molecular force fields [32] to relax the predicted structure. The per-residue distance difference tests (pLDDT) metrics are extracted from the b-factor column of the predicted structure PDB file, and only CA atoms are used for the calculation of the average pLDDT. The structure of EspN was derived from AlphaFold2 modeling. Based on the per-residue confidence score (pLDDT) (ranked 0: 84.0051, ranked 1: 83.8495, ranked 2: 82.5245, ranked 3: 81.8049, ranked 4: 81.5267) [33], we chose the ranked 0 model. The structure of EspS was derived from AlphaFold2 modeling. Based on the per-residue confidence score (pLDDT) (ranked 0: 86.3553, ranked 1: 85.9500, ranked 2: 85.3318, ranked 3: 85.3035, ranked 4: 84.9392), we chose the ranked 0 model.

We used the foldseek [34] server to perform a structural similarity search on the conserved regions of EspN and EspS. For FoldSeek, the “AlphaFold/Swiss-Prot v4” and “PDB100 20240101” databases are selected as the target [35]. “TM-align” [36] is used as the alignment mode, which gives higher precision than 3Di/AA. For EspN, we selected the structure corresponding to the sequence 673–1133 a.a. as the query. As a result, only one hit was found in PDB100. For EspS, we used the 146–291 a.a. sequence structure as the query and found 23 hits in UniProt–Swiss and 42 hits in PDB100. TM-score [37] is calculated using the similarity between protein structures. Finally, we found that PDBID: 6yhn [38] has a TM-score of 0.5157 with the predicted structure of EspN (673–1133 a.a.), and PDBID: 3eeb [39] has a TM-score of 0.5014 with the predicted structure of EspS (146–291 a.a.). Analyzing the section on structural comparisons revealed that 6yhn (530–700 a.a.) has a TM-score of 0.8601 with the predicted structure of EspN (673–843 a.a.), and PDBID: 3eeb (37–184 a.a.) has a TM-score of 0.7052 with the predicted structure of EspS (179–291 a.a.).

## 3. Results

### 3.1. EspN or EspS Is Translocated into Host Cells by T3SS

**Figure 1:**
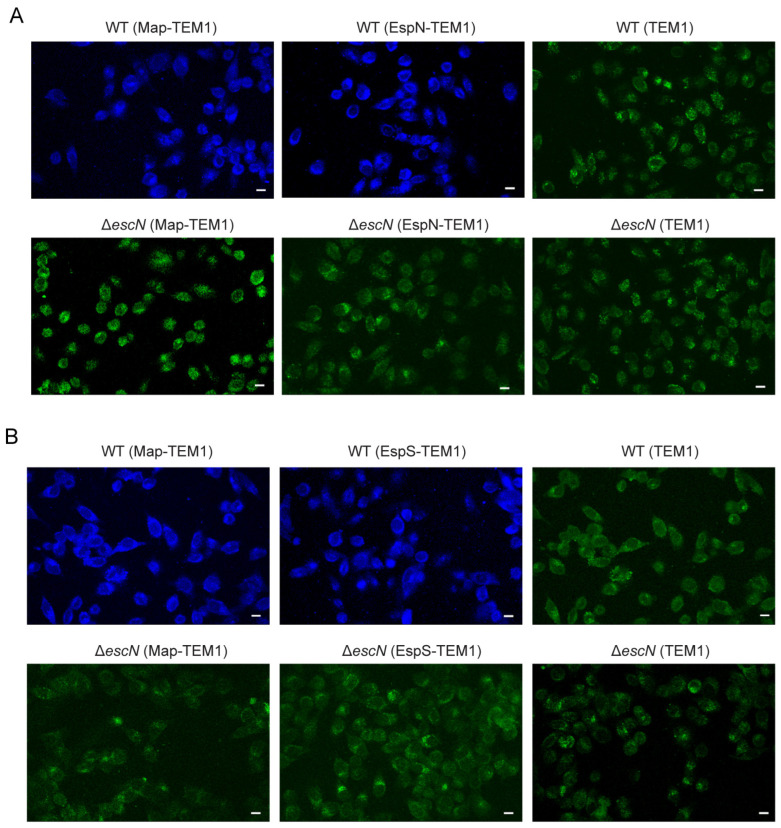
TEM1 (β-lactamase) translocation assay of EspN or EspS in HT-29 cells. (**A**) EspN-TEM1, Map-TEM1 fusion proteins, or TEM1 were expressed in *E. coli* O157:H7 WT or Δ*escN* strains. (**B**) EspS-TEM1, Map-TEM1, or TEM1 were expressed in *E. coli* O145:H28 WT or Δ*escN* strains. After infection with WT or Δ*escN* strains, HT-29 cells were loaded with CCF2/AM1 dye, and translocation was determined by a comparison of cleaved to uncleaved CCF2/AM1, which emits blue and green fluorescence, respectively. White bars indicate 10 μm.

The TEM-1-β-lactamase reporter system has been proven robust for assaying effector translocation into host cells [23]. In order to determine whether EspN or EspS was translocated into host cells by T3SS, we constructed fusion proteins of EspN or EspS and TEM-1 (β-lactamase). These constructs were introduced into both EHEC WT and Δ*escN*, a strain deficient in T3SS function. These strains were used to infect HT-29 cells loaded with the CCF2/AM substrate. HT-29 cells with no translocated TEM1, such as in the case of infection with EHEC-expressing TEM1, emitted green fluorescence at 520 nm (Figure 1A,B). In contrast, evident emission of a blue fluorescence at 450 nm was observed in cells infected for 4 h by WT EHEC expressing the EspN-TEM1 or EspS-TEM1 fusion protein. The blue fluorescence, generated by the β-lactamase-mediated cleavage of CCF2/AM and the consequent disruption of FRET, was indicative of translocated EspN-TEM1 (Figure 1A) or EspS-TEM1 (Figure 1B) fusion proteins. When the EspN-TEM1 or EspS-TEM1 fusion protein was expressed in the EscN mutant strain (Δ*escN*), infected HT-29 cells emitted only green fluorescence similar to that observed in control cells. Another known T3SS substrate, Map [40], was included in this assay as a positive control. All of these results indicate that EspN or EspS is translocated into host cells by T3SS.

### 3.2. Evolutionary Tree Analysis of EspN Revealed That Homologous Proteins of EspN Widely Distributed in Many Pathogens

**Figure 2:**
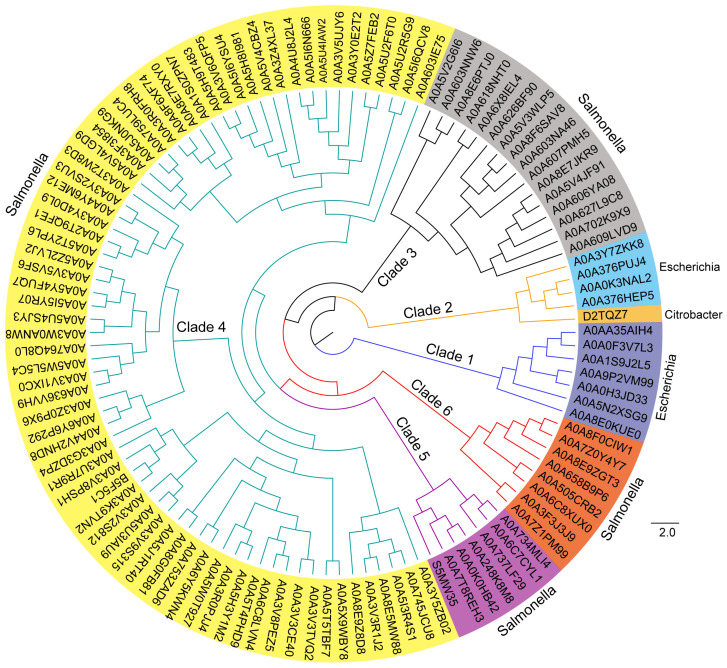
Evolutionary tree diagram of EspN and its homologs. Phylogenetic relationships of EspN and its homologs in the Uniprot-kb database. The species origin of the proteins was identified and indicated with different colors.

To determine the diversity and distribution of the bacterial effector EspN (1133 amino acids), a phylogenetic tree was constructed using the blastp algorithm within the Uniprot database. A total of 114 homologous proteins of EspN have been discovered, with an identity range of 35.57% to 100% (Figure 2 and Table S1). All homologs of EspN have been identified or predicted as T3SS-secreted effectors. More importantly, all homologs of EspN were with a conserved domain of unknown function (DUF4765) in the NCBI database, which consists of approximately 1100 amino acids. On the basis of evolutionary relationships, the homologous proteins of EspN were classified into six clades. There are six homologs of EspN in clade 1. All six homologs were distributed in pathogenic *E. coli* strains, including *E. coli* O26, *E. coli* O157, *E. coli* O145:NM, and *E. coli* 97.0246, and the sequences were substantially identical (99.74% to 100%). There are five homologous proteins in clade 2. Four homologs were distributed in *E. coli* strains, and one homolog, ROD_47811 (D2TQZ7), was in the *C. rodentium* strain, with an identity range of 36.36% to 39.03%. There are 15 homologous proteins in clade 3. All 15 homologs were distributed in *Salmonella* strains, including *Salmonella Newport*, *Salmonella enterica* subsp. *enterica* serovar Saintpaul, *S. enterica* subsp. *enterica* serovar Javiana, *S. enterica* I, *Salmonella anatum*, *Salmonella infantis*, and *Salmonella muenchen*, with an identity range of 35.89% to 36.70%. Clade 4 has the largest number of homologs, including 71 proteins. All 71 homologs were distributed in *Salmonella* strains, including *Salmonella senftenberg*, *S. enterica* subsp. *enterica* serovar, *S. enterica* I, *Salmonella agona*, *Salmonella derby*, *Salmonella Rubislaw*, and *Salmonella Montevideo*, with an identity range of 35.57% to 36.69%. There are eight homologous proteins in clade 5. All eight homologs were distributed in *Salmonella* strains, including *S. enterica* subsp. *diarizonae* serovar, *Salmonella diarizonae*, and *S. enterica* I, with an identity range of 37.75% to 37.84%. There are seven homologous proteins in clade 6. All seven homologs were distributed in *Salmonella* strains, including *S. enterica* subsp. *salamae* serovar, *Salmonella bongori*, with an identity range of 36.54% to 36.92%. In conclusion, the homologous proteins of EspN are widely distributed across multiple pathogenic bacteria, including *Salmonella*, *E. coli*, and *C. rodentium*, and the homologs from the same genus are generally in the same clade. These results suggest that EspN and its homologs represent an important class of T3SS effectors that serve as key virulence factors underlying the infection strategy of these pathogens. 

### 3.3. EspN and Its Homologues Share Conserved C-Terminal Sequences

**Figure 3:**
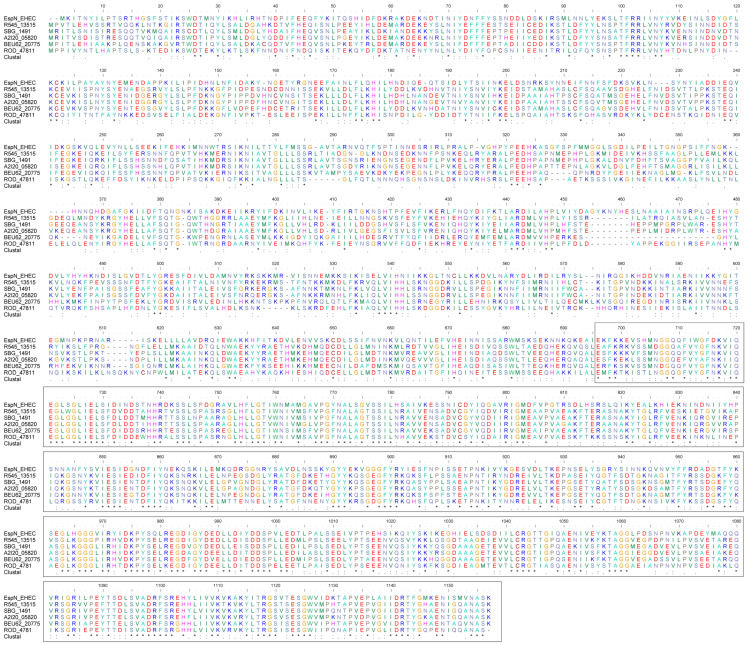
Multiple alignments of EspN and its homologs. Type III secretion system effector protein—EspN (from *E. coli* O157:H7 strain) was found to be homologous to five other proteins, including R545_13515 (from *S. enterica* subsp. *diarizonae* serovar strain), SBG_1491 (from *S. bongori* NCTC 12419 strain), A2I20_05820 (from *S. choleraesuis* strain), BEU62_20775 (from *S. newport* strain), ROD_47811 (from *C. rodentium* strain). The EspN family sequences were obtained from Uniprot in FASTA format and compared with multiple sequences using BioEdit software. The invariant bases are indicated with the same color. ‘.’ indicates that one of the following ‘weaker’ groups is fully conserved. ‘:’ indicates that one of the following ‘strong’ groups is fully conserved. ‘*’ indicates positions which have a single, fully conserved residue. Highly conserved regions of the sequence are boxed.

Further, one representative homologous protein from each clade was selected for multiple sequence comparisons in Figure 3. The selected homologous proteins included EspN in the EHEC O157:H7, R545_13515 in *S. enterica* subsp. *diarizonae* serovar (identity: 37.84%), SBG_1491 in *S. bongori* (identity: 37.59%), A2I20_05820 in *Salmonella choleraesuis* (identity: 36.20%), BEU62_20775 in *Salmonella newport* (identity: 36.59%), ROD_47811 in *C. rodentium* (identity: 39.03%). The C-terminal sequences of EspN and its homologous proteins (673–1133 a.a., 50.70 to 54.15%) have a significantly higher degree of identity than the N-terminal sequences (1–672 a.a., 28.03 to 30.27%). The results show that EspN and its homologous proteins share a sequence-conserved C-terminal region.

### 3.4. The Predicted 3D Structure of the Subsequences of EspN Shows SOME Similarity to the D4 Domain of CNF(Y)

**Figure 4:**
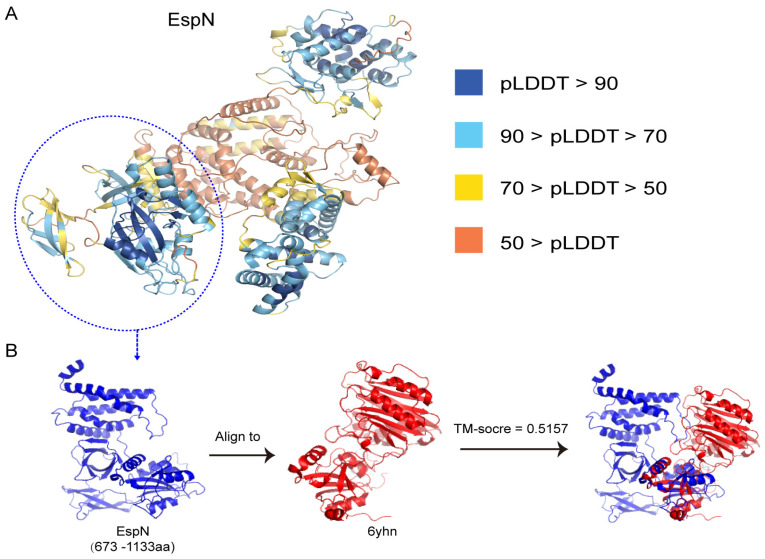
Protein structure prediction and analysis of EspN. (**A**) The 3D structure of EspN was generated using Alphafold2. AlphaFold produces a per-residue confidence score (pLDDT) between 0 and 100. Some regions with low pLDDT may be unstructured in isolation. (**B**) The foldseek server was used to perform a structural similarity search on the conserved C-terminal (673–1133 a.a.) of EspN. The predicted structures were analyzed and compared using PyMOL v2.5 software to identify the structural differences. The TM-score is calculated from the similarity between the protein structures. The structure of 6yhn has the highest TM score (0.5157).

Based on the protein sequences, we used Alphafold2 to predict the 3D structure of EspN. The predicted 3D structure showed high confidence scores (pLDDT value 83.6–85.3 on average) in three regions of EspN (19–206 a.a., 372–608 a.a., and 673–1133 a.a.) in Figure 4A. Interestingly, EspN (673–1133 a.a.) is the conserved C-terminal region of EspN. The well-defined 3D structure of EspN (673–1133 a.a., pLDDT value: 85.3) includes three subdomains connected by a loop region (Figure 4B). Consequently, we used foldseek to find similarly resolved experimental structures of EspN (673–1133 a.a.) from the PDB100 database [35]. The results showed that the structure of the bacterial cytotoxic necrotizing factor CNF(Y) (PDBID: 6yhn) has the highest TM score (0.5157) with the predicted structure of EspN (673–1133 a.a.) in Figure 4B. Structural comparisons show structural similarity between CNF(Y) and EspN for only a small portion of the sequence. The structure of CNF(Y) (530–700 a.a.) has a TM score of 0.8601, with the predicted structure of EspN (673–843 a.a.). CNFs are bacterial single-chain exotoxins that modulate cytokinetic/oncogenic and inflammatory processes through activation of host cell Rho GTPases [41,42]. To achieve this, they are secreted, bind surface receptors to induce endocytosis, and translocate a catalytic unit into the cytosol to intoxicate host cells. CNF(Y) consists of five domains (D1–D5). Analysis with PiSQRD [43] assigns domain boundaries to residues 1–22/135–424 (D1), 23–134 (D2), 425–529 (D3), 530–700 (D4), and 718–1014 (D5, deamidase domain). D1–3 act as export and translocation modules for the catalytic unit (D4–5). The role of the D5 domain is the targeting and modification of Rho GTPases. D4 interacts extensively with D5 in the crystal structure of the free D4–5 fragment [38]. EspN (673–843 a.a.) possesses structural similarity to the D4 domain (530–700 a.a.) of CNF(Y). Therefore, we hypothesize that the function of EspN (673–843 a.a.) has a similar function to the D4 domain of CNF(Y). The structure of EspN (844–1133 a.a.) is different from that of any other known structure, and the function of this region remains unknown. All results show that EspN has multiple structural domains and probably several unknown functional activities that need to be further investigated.

### 3.5. Evolutionary Tree Analysis of EspS Revealed That Homologous Proteins of EspS Widely Distributed in Many Pathogens

**Figure 5:**
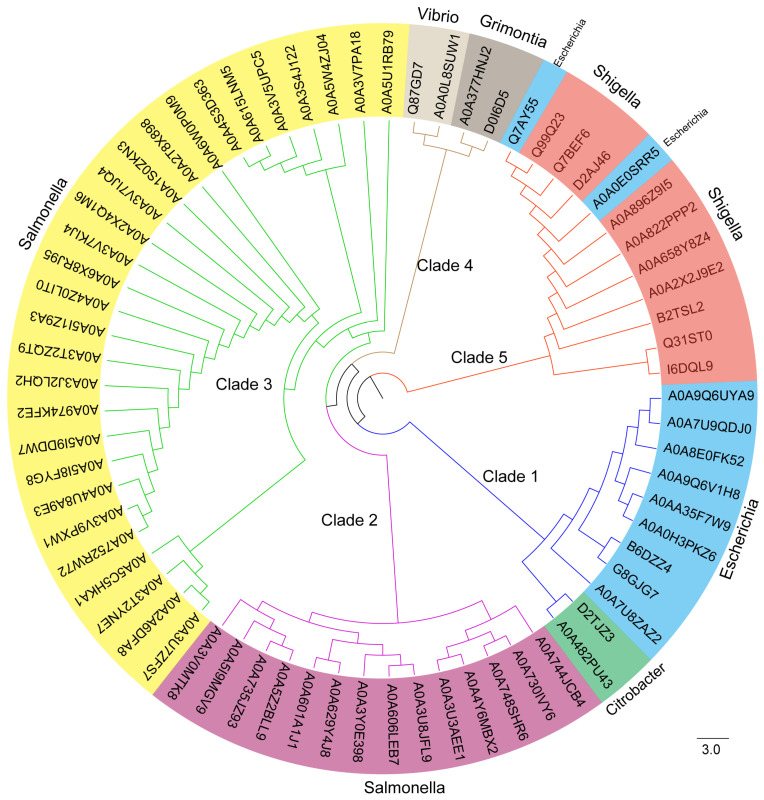
Evolutionary tree diagram of EspS and its homologs. Phylogenetic relationships of EspS and its homologs in the Uniprot-kb database. The species origin of the proteins was identified and indicated with different colors.

To determine the distribution of the bacterial effector EspS (291 amino acids), a phylogenetic tree was constructed using the blastp algorithm within the Uniprot database. Sixty-nine homologs of EspS have been discovered, with an identity range of 34.13% to 100% in Figure 5 and Table S2. On the basis of evolutionary relationships, homologous proteins were classified into five clades. There are 11 homologues of EspS in clade 1. Eight homologs were distributed in *E. coli* strains, including *E. coli* O145, *E. coli* 97.0246, *E. coli* O26, and *E. coli* O157:H7, with an identity range of 92.04% to 100%. Moreover, the other three homologs were found to be distributed in *Escherichia albertii* and *C. rodentium*, with an identity range of 63.92% to 71.48%. In comparison to *E. coli*, *E. albertii* and *C. rodentium* are closely related. There are 14 homologous proteins in clade 2. All 14 homologs were distributed in *Salmonella* strains, including *S. enterica*, *S. enterica* I, and *S. montevideo*, with an identity range of 36.05% to 38.08%. There are 29 homologous proteins in clade 3. All 29 homologs were distributed in *Salmonella* strains, including *S. enterica* subsp. *enterica* serovar, *S. enterica* I, and *Salmonella oranienberg*, with an identity range of 38.96% to 39.76%. There are four homologs in clade 4. Two homologs were distributed in *Vibrio* strains, with an identity range of 38.40%. The other two homologs were distributed in *Grimontia hollisae* strains, with an identity range of 37.18%. There are 12 homologous proteins in clade 5. Ten homologs were distributed in *Shigella* strains, including *S. flexneri* and *Shigella boydii*, with an identity range of 34.13%. Two homologs were distributed in *E. coli* strains, also with an identity range of 34.13%. In conclusion, the homologous proteins of EspS are widely distributed across multiple pathogenic bacteria, including *Salmonella*, *E. coli*, *Shigella*, *Vibrio*, *Grimontia*, and *C. rodentium*, and the homologs from the same genus are generally in the same clade. Additionally, all homologs of EspS have been identified or predicted as T3SS-secreted effectors. Given its wide distribution among pathogenic bacteria and its role as a T3SS effector, further studies on the potential contribution of EspS to bacterial virulence are warranted.

### 3.6. EspS and Its Homologues Share Conserved C-Terminal Sequences

**Figure 6:**
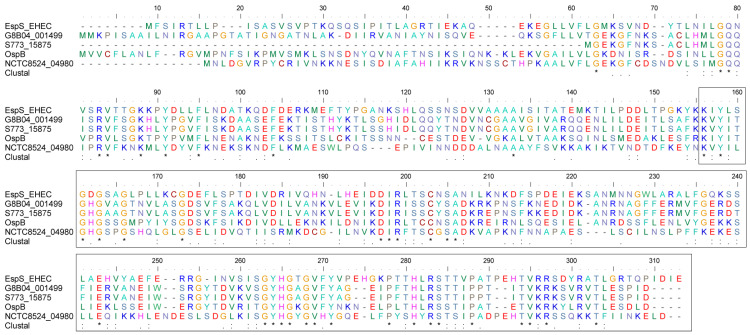
Multiple alignments of EspS and its homologs. Type III secretion system effector protein—EspS (from *E. coli* O145:H28 strain) was found to be homologous to four other proteins, including protein G8B04_001499 (from *S. enterica* strain), S773_15875 (from *S. rubislaw* strain), OspB (from *V. parahaemolyticus* strain), NCTC8524_04980 (from *S. flexneri* strain). The EspN family sequences were obtained from Uniprot in FASTA format and compared with multiple sequences using BioEdit software. The invariant bases are indicated with the same color. ‘.’ indicates that one of the following ‘weaker’ groups is fully conserved. ‘:’ indicates that one of the following ‘strong’ groups is fully conserved. ‘*’ indicates positions which have a single, fully conserved residue. Highly conserved regions of the sequence are boxed.

Further, we selected one representative homolog from each clade to go for multiple sequence comparisons in Figure 6. The selected proteins included EspS in the *E. coli* O145:H28 strain, G8B04_001499 in *S. enterica* (identity: 38.077), S773_15875 in *Salmonella rubislaw* (identity: 39.357), OspB in *Vibrio parahaemolyticus* (identity: 38.4), NCTC8524_04980 in *S. flexneri* (identity: 34.127). The results show that EspS and its homologous proteins share a sequence-conserved C-terminal region from amino acids 146 to 291, with an identity range of 44.16 to 44.90%.

### 3.7. The Predicted 3D Structure of the Subsequences of EspS Shows Some Similarity to That of the Vibrio cholerae RTX Cysteine Protease Domain

**Figure 7:**
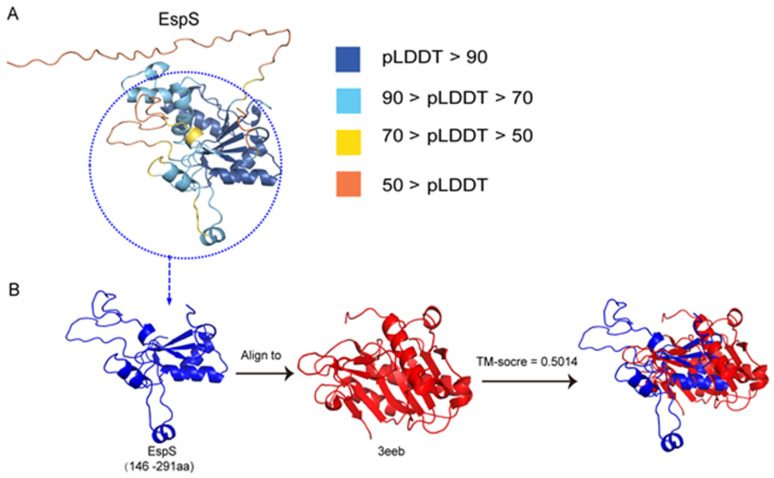
Protein structure prediction and analysis of EspS. (**A**) The 3D structure of EspS was generated using Alphafold2. AlphaFold produces a per-residue confidence score (pLDDT) between 0 and 100. Some regions with low pLDDT may be unstructured in isolation. (**B**) The foldseek server was used to perform a structural similarity search on the conserved C-terminal (146–291 a.a.) of EspS. The predicted structures were analyzed and compared using PyMOL software to identify the structural differences. The TM-score is calculated from the similarity between the protein structures. The structure of 3eeb has the highest TM score (0.5014).

Based on the protein sequences, we used Alphafold2 to predict the 3D structure of EspS. The predicted 3D structure showed low confidence scores (pLDDT value 56.2 on average) in the N-terminal of EspS (1–40 a.a.) that is composed of a loop, and high confidence scores (pLDDT value 81.5–84.0 on average) in EspS (41–291 a.a.) (Figure 7A). The conserved region of EspS (146–291 a.a.) is composed of a β-sheet consisting of four β-strands and several short helices. We also used foldseek to find similarly resolved experimental structures of the conserved C-terminal region of EspS from PDB100, which showed that the structure of the *V. cholerae* RTX cysteine protease domain (CPD) (PDBID: 3eeb) has the highest TM score (0.5014) in Figure 7B. Structural comparisons show that the structure of the RTX CPD (37–184 a.a.) has a TM score of 0.7052 with the predicted structure of EspS (179–291 a.a.). *V. cholerae* RTX (repeats in toxin) is an actin-disrupting toxin that is autoprocessed by the internal CPD [39]. These results provide important clues to the possible function of EspS and its potential role in bacterial infections.

## 4. Discussion

Type III secretion system (T3SS) effectors are key virulence factors that play a pivotal role in the infection strategy of numerous clinically important Gram-negative pathogens, including *S. enterica*, *Shigella* spp., enteropathogenic and enterohemorrhagic *E. coli* (EHEC, EPEC), and their murine counterpart, *C. rodentium* [44]. In recent years, numerous studies have elucidated the structural and functional characteristics of a multitude of effector molecules, including Map, Tir, the Nle family (NleH, NleD, NleB, NleC, and NleE), and the Esp family (EspT, EspM, EspG, EspH, EspW, and EspF) [10]. There are still some predicted effector proteins whose functional information is completely unknown, such as EspN and EspS. In this study, we initially performed the TEM1 (β-lactamase) translocation assay, which confirmed that the two predicted effectors, EspN and EspS, were translocated into host cells in a T3SS-dependent manner. To elucidate the diversity and function of EspN and EspS, we analyzed their sequence characteristics and distribution in pathogenic bacteria. The results of the phylogenetic analysis have shown that homologs of EspN and EspS are widely distributed among pathogenic bacteria, including *E. coli*, *Salmonella*, and other pathogens that possess type III secretion systems [45]. These findings suggest that EspN and EspS represent two important classes of effectors involved in the infection of numerous pathogens. Sequence analysis revealed that the homologous proteins of EspN and EspS from the same genus of bacteria had a high degree of similarity, and the majority of them belonged to the same clade. The results suggest that EspN and EspS were acquired during the early stages of bacterial evolution. Concurrently, we discovered that both EspN and EspS have highly conserved C-terminal sequences. These results suggest that the core domains encoded by their C-terminal sequences deserve further investigation.

Alphafold2 is widely used for protein structure prediction [31,46,47]. AlphaFold2 has been utilized to predict the 3D structure of almost the entire human proteome (98.5% of human proteins). The resulting dataset encompasses 58% of residues with a confident prediction, with a subset (36% of all residues) exhibiting exceptionally high confidence [48,49,50,51]. However, few studies have been conducted on structure prediction and functional analysis of pathogen effectors using Alphafold2 and other similar software. In this study, Alphafold2 was used to predict the structures of two effectors, EspN and EspS, which are widely distributed in *E. coli*, *Salmonella*, and other pathogenic enterobacteria. The results showed that the predicted structures of the C-terminal conserved regions of EspN (673–1133 a.a.) and EspS (146–291 a.a.) have some similarity to the structures of currently known functional proteins. EspN (673–843 a.a.) has a high degree of structural similarity to the D4 (530–700 a.a.) domain of CNF(Y) (PDBID: 6yhn) [38]. The D4 domain interacts extensively with the D5 domain (718–1014 a.a.), which targets and modifies Rho GTPases. However, the exact function of the D4 domain is not yet known. Further investigation into the significance of the EspN (673–843 a.a.) or D4 domain of CNF(Y) is warranted. More importantly, EspN (844–1133 a.a.) has unique structural features that are inconsistent with all currently known protein structures and warrant further investigation. The structure of the C-terminal conserved region of EspS (146–291 a.a.) shows some similarity to the structure of the *V. cholerae* RTX cysteine protease domain (PDBID: 3eeb). The *V. cholerae* RTX is an actin-disrupting toxin that is autoprocessed by the internal cysteine protease domain [39]. This structural information about EspN and EspS provides important clues for parsing their functions. The T3SS is critical for the virulence of Gram-negative pathogens, and the effectors delivered by the T3SS target many fundamental processes within the infected host cell [10]. The structural similarity to the important virulence factors CNF(Y) and RTX suggests that EspN and EspS may be two classes of undiscovered virulence factors that may play important roles in the pathogenesis of bacterial infections.

## 5. Conclusions

In conclusion, we have systematically analyzed the diversity, distribution, and structure of two T3SS effectors of unknown function, EspN and EspS. Phylogenetic tree analysis revealed that homologs of EspN and EspS are widely distributed in Gram-negative pathogens, including *Escherichia*, *Salmonella*, and *Citrobacter*. Multiple sequence alignment revealed that both EspN and EspS families share conserved C-terminal regions of unknown function. The results of the structure prediction indicated that partial sequences of the conserved C-terminal regions of EspN and EspS show some similarity to the currently known functional proteins CNF(Y) and RTX, respectively. All results suggest that EspN and EspS, delivered by the T3SS, target a multitude of fundamental processes within the infected host cell, and our findings provide important clues to the possible function of EspN and EspS. With increasing knowledge of the distribution and structure prediction of bacterial effectors, a future challenge will be to investigate how these effectors interact with host cells and contribute to the course of an infection, which will be important in elucidating bacterial pathogenesis.

## Data Availability

All data presented are available in the manuscript.

## Supplementary Materials

The following supporting information can be downloaded at: https://www.mdpi.com/article/10.3390/genes15101250/s1, Table S1: Bioinformatics analyses of EspN, Table S2: Bioinformatics analyses of EspS.
